# Assessing mitochondrial heteroplasmy using next generation sequencing: A note of caution

**DOI:** 10.1016/j.mito.2018.08.003

**Published:** 2019-05

**Authors:** Mauro Santibanez-Koref, Helen Griffin, Douglass M. Turnbull, Patrick F. Chinnery, Mary Herbert, Gavin Hudson

**Affiliations:** a Institute of Genetic Medicine, International Centre for Life, Central Parkway, Newcastle upon Tyne NE1 3BZ, UK; b The Wellcome Centre for Mitochondrial Research, Newcastle University, Medical School, Framlington Place, Newcastle upon Tyne NE2 4HH, UK; c MRC Mitochondrial Biology Unit, Wellcome Trust/MRC Building, Cambridge Biomedical Campus, Hills Road, Cambridge CB2 0XY, UK; d Newcastle Fertility Centre, International Centre for Life, Central Parkway, Newcastle upon Tyne NE1 3BZ, UK

**Keywords:** Mitochondrial DNA, Next-generation sequencing, Heteroplasmy, Bioinformatic analysis

## Abstract

The mitochondrial genome has recently become the focus of several high-impact next-generation sequencing studies investigating the effect of mutations in disease and assessing the efficacy of mitochondrial replacement therapies.

However, these studies have failed to take into consideration the capture of recurring translocations of mitochondrial DNA to the nuclear genome, known as nuclear mitochondrial sequences (NUMTs), continuing to align sequence data to the revised Cambridge reference sequence alone.

Here, using different mtDNA enrichment techniques and a variety of tissues, we demonstrate that NUMTs are present in sequence data and that, dependent upon downstream analysis, are at a level which affects variant calling.

## Highlights

• Translocations of mtDNA to the nDNA genome are commonplace and present a challenge when performing next-generation-sequencing experiments aimed at identifying mtDNA heteroplasmy.
• Accurate next generation sequencing of mtDNA is affected by both target enrichment and downstream bioinformatic analysis strategy.
• NUMTs can affect heteroplasmy calling, but cannot wholly explain low-level sequencing artefacts.

## 1 Introduction

Compared to nuclear DNA, mitochondrial DNA (mtDNA) is highly mutable (Hodgkinson and Eyre-Walker, 2011) and variants can be present in all (homoplasmy) or a proportion (heteroplasmy) of the molecules (Stewart and Chinnery, 2015). Mitochondrial DNA-like sequences in the nucleus (NUMTs) can interfere with the detection of heteroplasmy. For example, human chromosome 8 contains almost an entire mtDNA sequence inserted into the first intron of *SDC2 (**Dayama* et al., *2014**)*. This may yield misleading results in relation to heteroplasmy levels and highlights a need for greater rigor in the analysis of mtDNA sequence data, particularly as recent studies indicate that on average human individuals carries approximately 750 NUMTs, ~4 of which are typically unique to each individual (Dayama et al., 2014; Genomes Project et al., 2015).

With some exceptions, diseases caused by pathogenic mtDNA mutations are characterised by the co-existence of wildtype and mutated mtDNA. Crucially, the severity of disease symptoms associated with pathogenic mutations is determined by the relative levels of wild type and mutated mtDNA. Thus, accurate quantification of heteroplasmy, i.e. determination of allele frequencies at sites showing heteroplasmic variation, is critically important in the diagnosis, treatment and genetic counselling of mtDNA diseases (Stewart and Chinnery, 2015). For example, for diseases such as myoclonic epilepsy and ragged-red fibres, in vitro studies have shown that the frequency of deleterious variant must exceed a threshold (typically 60–80%) before a biochemical deficit manifests, therefore accurate quantification is important for genetic diagnosis (Boulet et al., 1992; King and Attardi, 1989). Importantly, because of the way in which mtDNA variants segregate during development of the female germ line, women who are heteroplasmic for pathogenic mtDNA mutations can produce eggs with widely varying mutation loads (Shoubridge and Mitochondrial, 2000). This makes it very difficult to predict the risk of disease in their children (Wallace and Chalkia, 2013). Thus, accurate quantification of heteroplasmy is therefore also essential for prenatal diagnosis and, more recently, is critical for reproductive technologies such, such as pre-implantation genetic diagnosis (PGD) and mitochondrial donation/replacement procedures such as pronuclear or spindle transfer (Richardson et al., 2015).

In recent years several studies have utilised next generation sequencing (NGS) to investigate heteroplasmic mtDNA variation, assess the likely efficacy of mitochondrial replacement therapy in disease prevention (Kang et al., 2016a; Yamada et al., 2016), and investigate the effect of mtDNA variation on cell biology [12] and in human disease (Dolle et al., 2016; Rygiel et al., 2016; van der Walt et al., 2012). The materials analysed in such investigations typically range from single cells to tissue homogenates (Dolle et al., 2016; Rygiel et al., 2016; van der Walt et al., 2012). In the vast majority of studies, one of three strategies are employed to isolate and enrich mtDNA for sequencing: several overlapping PCR amplicons (typically 100–2000 base-pairs long) (Payne et al., 2011; Payne et al., 2015), long-range PCR (typically one or two overlapping large amplicons) (Kang et al., 2016a; Rygiel et al., 2016; van der Walt et al., 2012) or more recently, commercially available mtDNA enrichment kits (typically relying on multiple displacement amplification producing series of overlapping sequence fragments) (Ancora et al., 2017; Marquis et al., 2017). Earlier work amplifying the mtDNA genome as one large amplicon showed promise (Zhang et al., 2012), unfortunately this does not appear to have been widely adopted. Enriched mtDNA is sequenced and typically mapped to a mtDNA reference sequence such as the revised Cambridge Reference Sequence (rCRS, NM_012920.1), which is predominantly used to maintain variant position numbering consistency (Ancora et al., 2017; Dolle et al., 2016; Kang et al., 2016a; Kang et al., 2016b; Marquis et al., 2017; Payne et al., 2011; Payne et al., 2015; Rygiel et al., 2016; van der Walt et al., 2012; Yamada et al., 2016).

Given the importance of accurate heteroplasmy assessment for clinical and research purposes, we have systematically investigated the effect of the sample type (pooled cells and tissue homogenates), enrichment strategy and reference sequence on heteroplasmy assessment using the same bioinformatics pipeline. In addition, to gain insight into the impact of NUMTs on heteroplasmy assessment, we mapped mtDNA sequence data either to both the mtDNA reference sequence (rCRS or NM_012920.1) in isolation and to the combined nuclear (hg19) and mitochondrial (rCRS or NM_012920.1) genomes (hereafter referred to as simply hg19). Our findings indicate that the reference sequence choice has a significant effect on mtDNA variant calls, and that the results are also dependent on the isolation and enrichment steps used.

## 2 Materials and methods

mtDNA was extracted and enriched for next-generation sequencing from a range of tissues using established methodology (Supplementary materials). Next-generation sequencing was performed as per manufactures guidelines (Supplementary materials). Downstream bioinformatic analysis was performed as described previously (Coxhead et al., 2016; Payne et al., 2011; Payne et al., 2015) (and including Supplementary materials). Raw data and analytical pipeline are available at: https://doi.org/10.5281/zenodo.1157051

## 3 Results

**Fig. 1:**
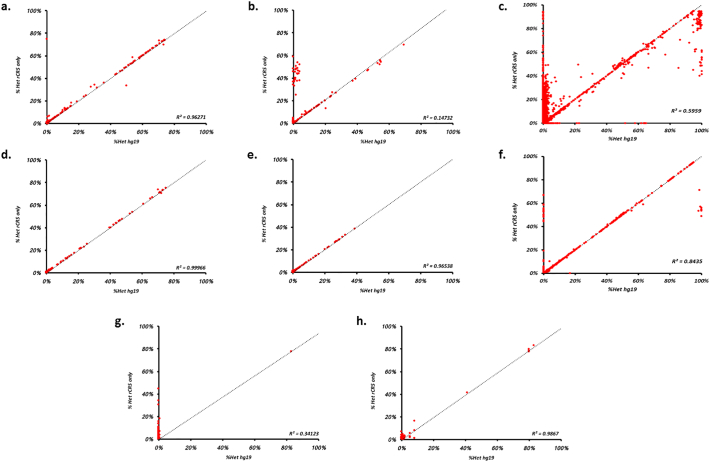
Comparison of variant heteroplasmy level derived from alignment to either hg19 (x-axis) or the rCRS in isolation (y-axis) for different starting materials and enrichment procedures. Where, a) Pooled cells A; 2 amplicon LR-PCR, b) Pooled cells B; 9 amplicon PCR, c) Pooled cells C; 180 amplicon PCR, d) Tissue Homogenate; 2 amplicon LR-PCR, e) Tissue homogenate; 9 amplicon PCR, f) Tissue homogenate; 180 amplicon PCR, g) CSF; MDA and h) CSF; 2 amplicon LR-PCR. The dotted line indicates the x/y reference line, i.e. where rCRS heteroplasmy = hg19 heteroplasmy.

**Fig. 2:**
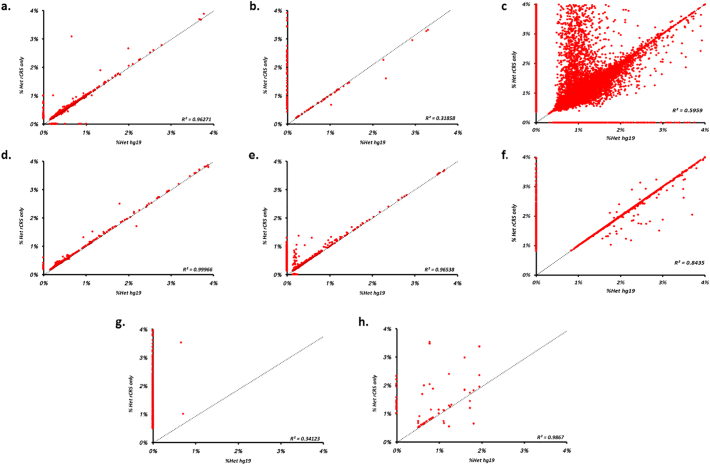
Rescaled representation showing the effect of aligning to either hg19 (x-axis) or the rCRS in isolation (y-axis) for frequencies below 4%. Where, a) Pooled cells; 2 amplicon LR-PCR, b) Pooled cells; 9 amplicon PCR, c) Pooled cells; 180 amplicon PCR, d) Tissue Homogenate; 2 amplicon LR-PCR, e) Tissue homogenate; 9 amplicon PCR, f) Tissue homogenate; 180 amplicon PCR, g) CSF; MDA and h) CSF; 2 amplicon LR-PCR. The dotted line indicates the x/y reference line, i.e. where rCRS heteroplasmy = hg19 heteroplasmy.

To test the effect of different isolation and enrichment steps, we compared heteroplasmy levels, i.e. the relative frequencies of the variants calls, using both rCRS-only and hg19 (which includes the rCRS reference) reference sequences (Fig. 1). To highlight the effect on low level variation, the region representing relative variant frequencies below 4% is expanded in Fig. 2. Our data show that reference sequence choice has a significant effect on mtDNA variant calls, particularly those at low level, and that the results are also dependent on the isolation and enrichment steps used.

Using a two-amplicon long-range PCR approach to isolate and enrich mtDNA from either pooled cells or homogenate tissue, we found a high correlation in variant heteroplasmy when aligned to either rCRS alone or to the combined rCRS and hg19 reference sequences (R^2^ = 0.963 and R^2^ = 0.999 respectively, Figs. 1a &1d, and Fig. 2a and d); with the heteroplasmy of 96.3% (of 2486 variants) and 97.7% (of 260 variants) variants differing by <0.5% (Supplementary Table 1).

We found that as amplicon size decreases the number of variants with discrepant frequencies increases (Supplementary Table 1 and, Fig. 1, Fig. 2). In the majority of cases, the frequency obtained using the rCRS-only as a reference is higher than that derived using hg19 (typically a ~2 to 50% increase). This appears to be influenced by both the initial enrichment procedure as well as the starting material. Notably, mtDNA enrichments from pooled cells (Fig. 1b and c) compared with enrichment from tissue homogenate shows significantly higher numbers of variants with higher frequencies in analyses using alignment to the rCRS only. Using 9 amplicons and pooled cells as starting material, the frequency difference exceeded 0.5% for 5.5% of the variants while this was observed for only 0.2% of the variants when tissue homogenate was used (*p* < .01). For a 180 amplicon strategy, the corresponding values were 3.0% and 0.4% (p < .01, Supplementary Table 1). It is not clear what is causing this phenomenon, although it is likely that differences in starting material, which is known to affect PCR amplification efficiency and accuracy in low-template reactions is to blame (Akbari et al., 2005).

The most extreme discrepancies we observed are putative variants that were only called when using one reference but not the other (i.e. rCRS-only versus hg19). In most cases, alignment to the rCRS-only detected heteroplasmic sites that could not be detected when aligning to hg19 (Supplementary Table 1). These variants are typically low level (Fig. 2 and Supplementary Table 1), however some achieve frequencies above 20% and up to 100% (Fig. 1b and c). Furthermore, the number of variants appears to be influenced by both starting material and amplification strategy, generally increasing in frequency as amplicon size decreases (Supplementary Table 1). Trimming the reads to remove primer sequences used in the 180-amplicon enrichment reduced the overall variant count (Supplementary Fig. 1), however this does not diminish the number rCRS/hg19 discrepancies (Supplementary Table 3).

Analysis of sequence data generated from multiple displacement amplification (MDA, Fig. 1g & 2g), showed very poor agreement between the results obtained using rCRS-only and hg19. By contrast, this was not seen when the same samples were amplified using a two-primer long range PCR strategy (Fig. 1h & 2h). Similar to our other experiments, sequence alignment to the rCRS alone following MDA resulted in a high proportion (99% or 966 of all variants observed, =976) of variants present only when aligning to the rCRS, which were subsequently removed when the data was aligned to hg19.

## 4 Discussion

Our results show discrepancies between the variant calls obtained using different reference genomes. It is possible that including the nuclear genome in the reference excludes genuine mitochondrial variants, where the variation appears more similar to nuclear sequences than to the mitochondrial sequence included in the reference. However, the reduction in the number of discrepancies using larger amplicons indicates that a large proportion of the discrepancies reflect amplification on nuclear sequence. Given the stoichiometry between mitochondrial and single copy nuclear DNA this is likely to result in putative variants present at a low frequency. Their number would also be expected to increase when the complete mitochondrial genome is assessed using a set of short amplicons, since it is more difficult to avoid placing some of these within NUMTs and because of increased ambiguity when mapping shorter sequences. These considerations are consistent with our observations, where there is very little discrepancy in experiments using a two amplicon approach (see Fig. 1, Fig. 2) as amplicon sizes are beyond the typical NUMT size (~100–6000 bp) (Bensasson et al., 2003).

**Fig. 3:**
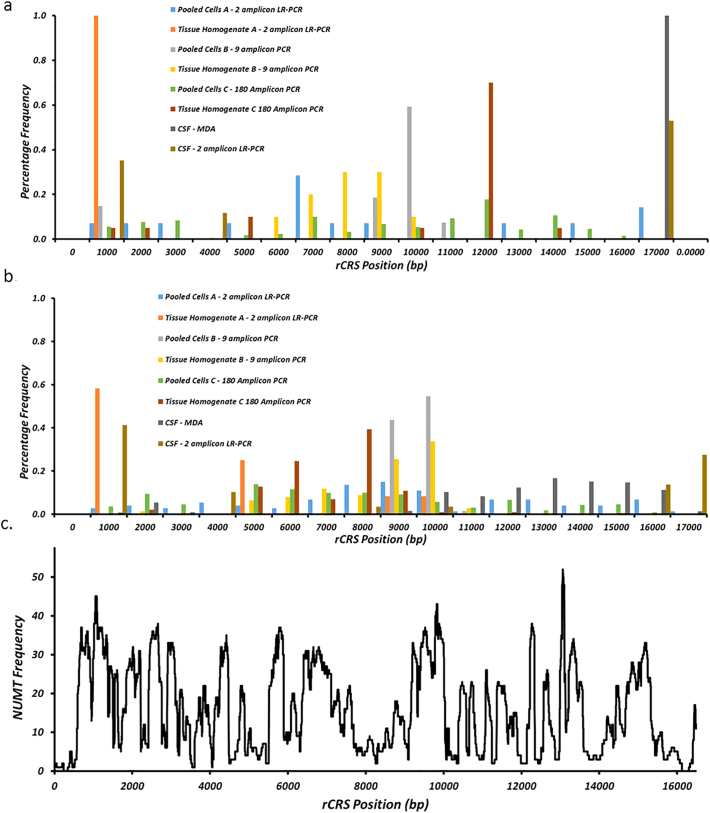
Frequency histogram showing the distribution of observed heteroplasmic variation relative to the position of known NUMT interference. Where a) Location of variants where the inferred frequency is higher when aligning to rCRS than to hg19), b) is the distribution of putative variants observed only when aligning to the rCRS (i.e. not observed when alignments hg19 are used) and c) is the frequency of NUMTs coverage per mtDNA base pair.

Although differences between tissue type and enrichment strategy are known to affect the number of variants detected, in particular low frequency variants (Dapprich et al., 2016), our findings indicate that the choice of reference sequence seems to have a considerably large impact. The location of variants showing discrepancies in frequencies between analyses using different reference appears non-random, clustering around the D-loop and between 5000 and 10,000 bp (Fig. 3), and appears independent of amplification strategy. However the association between variant position and primer binding site location did no achieve statistical significance.

Thus, the most likely explanation for this is the co-amplification and subsequent sequencing of NUMTs. However we found no significant correlation between variant position and mtDNA positions which are covered by known NUMTs (Supplementary Table 3). NUMT/mtDNA mean similarity is estimated as ~86% (standard deviation 4.1%, Supplementary Fig. 2), however we found no correlation between percentage similarity and mtDNA variant positions. Taken together, this indicates that other factors, such as the choice of starting material, also contribute to inaccuracy in the assessment of mtDNA heteroplasmy.

## 5 Conclusion

In conclusion, the accurate detection and quantification of mitochondrial heteroplasmy is dependent upon the initial enrichment strategy used, but more importantly on the reference that is employed in the downstream bioinformatic analysis. In particular, care should be taken interpreting heteroplasmic variants occurring within the D-loop and between 5000 and 10,000 base pairs of the mtDNA. If less specific enrichment strategies are used, i.e. small amplicons or MDA, then alignment should be compared to the combined nuclear and mitochondrial genomes (i.e. hg19). However, if a more specific enrichment strategy, i.e. multiple, longer, or one amplicon (Zhang et al., 2012), is used then alignment to the rCRS in isolation may suffice.

## Appendix A Supplementary data

Supplementary material Image 1
