# Supplementary material for: Assessing mitochondrial heteroplasmy using next generation sequencing: A note of caution
Source: Mitochondrion. 2019 May;46:302–6. doi: 10.1016/j.mito.2018.08.003 (PMC6509278; doi:10.1016/j.mito.2018.08.003)
Supplement: Supplementary file 1 — Supplementary material [file mmc1.docx]

**Supplementary Materials**

All cell and tissue samples and sequencing data existed as part of ongoing studies within the Welcome Trust Centre for Mitochondrial Research (Newcastle University, UK).

*Sample cohorts and corresponding enrichment strategy:*

**Cohort 1** (designated ‘**pooled cells A**’) is the result of sequencing n=25 embryonic stem cell line samples, enriched using a 2-primer long range PCR strategy (detailed below). Each cell pellet contained approximately 10^-6^ cells from which total DNA was extracted using established cell lysis methods.^1^

**Cohort 2** (designated ‘**tissue homogenate A**’) is the result of sequencing n=25 frontal cortex tissue homogenate samples, enriched using a 2-primer long range PCR strategy (detailed below). Each tissue sample was approximately 100 mg from which total DNA was extracted using a Qiagen Micro DNA tissue Kit (Qiagen, Netherlands).

**Cohort 3** (designated ‘**pooled cells B’**) is the result of sequencing n=5 embryonic stem cell line samples, enriched using a 9-primer PCR strategy (detailed below). Each cell pellet contained approximately 10^-6^ cells from which total DNA was extracted using established cell lysis methods.^1^

**Cohort 4** (designated ‘**tissue homogenate B**’) is the result of sequencing n=25 frontal cortex tissue homogenate samples, enriched using a 9-primer long range PCR strategy (detailed below). Each tissue sample was approximately 100 mg from which total DNA was extracted using a Qiagen Micro DNA tissue Kit (Qiagen, Netherlands).

**Cohort 5** (designated ‘**pooled cells C**’) is the result of sequencing n=25 cheek buccal epithelial cell samples, enriched using a 180-primer PCR strategy (detailed below). Each cell pellet contained approximately <10^-6^ cells from which total DNA was extracted using established cell lysis methods.^1^

**Cohort 6** (designated ‘**tissue homogenate C**’) is the result of sequencing n=25 frontal cortex tissue homogenate, enriched using a 180-primer PCR strategy (detailed below). Each tissue sample was approximately 100 mg from which total DNA was extracted using a Qiagen Micro DNA tissue Kit (Qiagen, Netherlands).

**Cohort 7** (**designated CSF**) is the result of sequencing n=5 CSF samples known to harbour high levels of mtDNA, but low levels of nDNA.^2^ Each sample was enriched using MDA (detailed below), with a corresponding 2-primer long range PCR control for comparison (detailed below). Each CSF sample ~500ul from which DNA was extracted using a Qiagen Micro DNA tissue Kit (Qiagen, Netherlands). In total 131 tissue samples were used in this experiment.

*mtDNA Enrichment*

2-primer long range and 9-amplicon PCR based enrichment of mtDNA was performed using previously reported methods.^3^ In both instances, amplicons were quantified using an Agilent 2100 Bioanalyzer™ (Agilent, UK) and pooled in equimolar sample pools. Each sample pool was subsequently purified by AMPure XP beads (Beckman Coulter Life Sciences, Buckinghamshire, UK) for library preparation.

180-amplicon sequencing was outsourced to Source BioScience™ (Nottingham, UK). Briefly, mtDNA was enriched using Access Array Integrated Fluidic Circuits™ (Fluidigm, UK), approximately 180 Amplicons were designed (150 to 200bp) to cover the entire mtDNA. 48 PCR reactions were performed on each sample. PCR products were then pooled in equal volumes to create one PCR product library which were then purified by AMPure XP beads (Beckman Coulter Life Sciences, Buckinghamshire, UK). PicoGreen fluorimetry was used to quantify the PCR product library prior to loading for DNA sequencing.

Multiple displacement amplification was performed using a Qiagen REPLI-g Mitochondrial DNA kit as per manufacturer’s guidelines.

*NGS sequencing:*

All samples were prepare and sequenced using Illumina library preparation and sequencing kits (Illumina, Ca, USA). Cohorts 1, 2, 3, 4, and 7 were sequenced using an Illumina MiSeq (Illumina, Ca, USA) and cohorts 5 and 6 were sequenced using an Illumina HiSeq2500 (Illumina, Ca, USA) both according to manufacturer’s protocols. Raw FASTQ reads of all samples were filtered to QV>30 prior to export for analysis.

*Bioinformatic Pipeline:*

All samples were subjected to the same bioinformatic analysis. Briefly, raw FASTQ reads were filtered using FASTQ (v0.11.2). Filtered reads were aligned to both the rCRS (NM_012920.1) and the human reference genome (hg19) using BWA (v0.7.12). Duplicate reads were removed using Picard (v1.130). Subsequent variant calling was performed using VarScan (v2.3.7). Coverage of the mtDNA was generated for all samples and samples <99% coverage were removed from further analysis (n=1 CSF sample generated using MDA).

FASTQ files generated using the 180-amplicon enrichment strategy (Source BioScience,™ Nottingham, UK) were subjected to primer trimming using cutadapt v1.14. Briefly, exact primer sequences were trimmed from the 5’ end of each FASTQ read. Subsequently trimmed FASTQ files were then subjected to the same bioinformatic pipeline as above. Supplementary Figure 1 compares heteroplasmies identified using both rCRS and hg19 alignments between non-trimmed and trimmed data.

In all instances, heteroplasmic variants are defined as >0.0%, but < 95%. When comparing between alignment types, skews were defined as >0.5% different between alignments and *‘only in X’* variants were defined as >0.5% on one reference (i.e. rCRS) and 0.0% on the other (i.e. hg19) and vice versa. Heteroplasmic variant calls, with comparative rCRS:hg19 heteroplasmies are available in Supplementary Table 2.

*NUMT analysis:*

Nuclear mitochondrial sequence (NUMT) information was taken from Hazkani-Covo *et al* (2010),^4^ data available at https://sourceforge.net/projects/dmcrop/files/human%20

NUMTs%20database/. All mtDNA positions (n=8010) known to be influenced by a NUMT (n=38103) were used to construct a frequency histogram of NUMT coverage per mtDNA bases pair (Figure 3c). The frequency distribution of NUMTs was compared to the frequency distribution of positively skewed and *‘only in X’* heteroplasmic variants by Spearman’s non-parametric correlation (SPSS v15.5). NUMT/mtDNA similarity is expressed as percentage simialrty between NUMT (reference hg19) and mtDNA (reference rCRS, NM_012920.1) Data was extracted from Hazkani-Covo *et al* (2010).^4^

*Supplementary References:*

*1. Payne BA, Wilson IJ, Hateley CA, Horvath R, Santibanez-Koref M, Samuels DC, Price DA, Chinnery PF. Mitochondrial aging is accelerated by anti-retroviral therapy through the clonal expansion of mtDNA mutations. Nat Genet. 2011 Jun 26;43(8):806-10.*

*2. Pyle A, Brennan R, Kurzawa-Akanbi M, Yarnall A, Thouin A, Mollenhauer B, Burn D, Chinnery PF, Hudson G. Reduced cerebrospinal fluid mitochondrial DNA is a biomarker for early-stage Parkinson's disease. Ann Neurol. 2015 Dec;78(6):1000-4.*

*3. Coxhead J, Kurzawa-Akanbi M, Hussain R, Pyle A, Chinnery P, Hudson G. Somatic mtDNA variation is an important component of Parkinson's disease. Neurobiol Aging. 2016 Feb;38:217.e1-217*

*4. Hazkani-Covo E, Zeller RM, Martin W. Molecular poltergeists: mitochondrial DNA copies (numts) in sequenced nuclear genomes. PLoS Genet. 2010 Feb 12;6(2):e1000834.*

**Supplementary Figure 1.** Comparison of heteroplasmic variant frequencies estimated using either untrimmed reads (left panels, blue) or after trimming of the amplification primers (right panels, red). Enrichment was achieved using 180 amplicons. Where, a) and c) pooled cells; b and d) Tissue homogenate; c) and d) show details for frequencies below 4%. N= number of observed heteroplasmies.

**
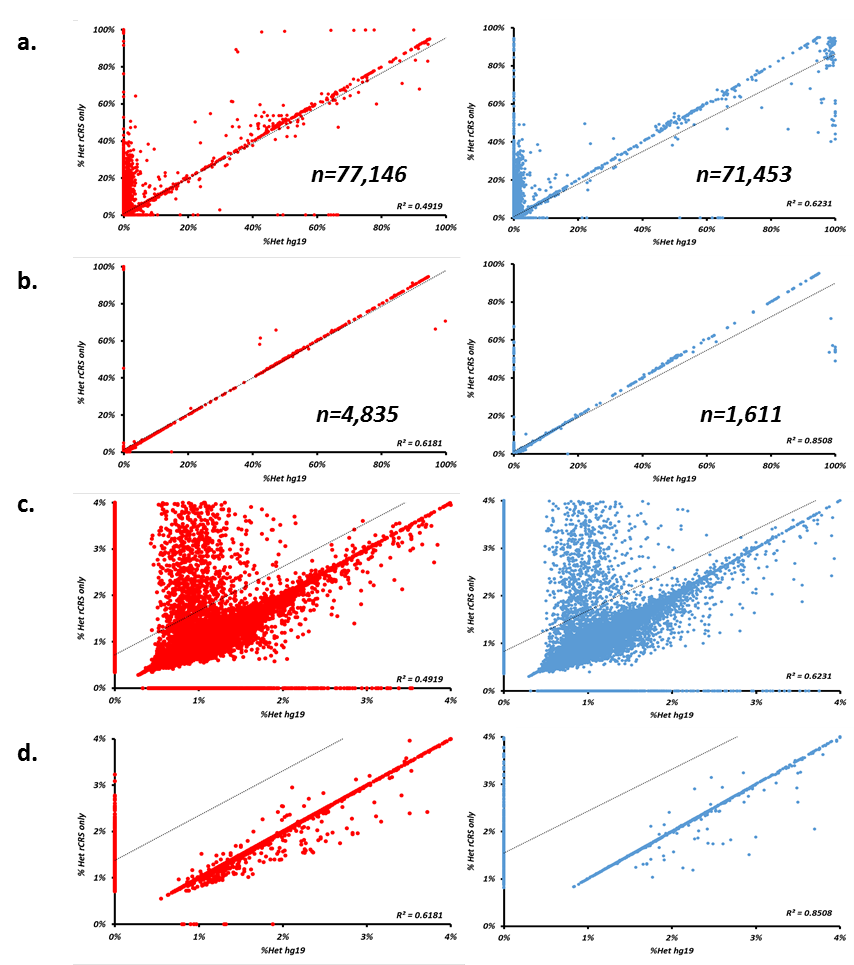
**

**Supplementary Figure 2.** The first panel shows the mean percentage similarity between NUMT and mtDNA sequence generated using BLASTN (data extracted from Hazkani-Covo et al. 2010),^4^ where black dots are mean similarity and whiskers are the percentage range). Mean percentages are shown binned in 200bp segments. Mean NUMT/mtDNA similarity is 86% with little deviation along the mtDNA sequence (Standard deviation 4.1%, Hazkani-Covo et al. 2010).

The second, third and fourth panels reproduce Figure 2 for reference.

**
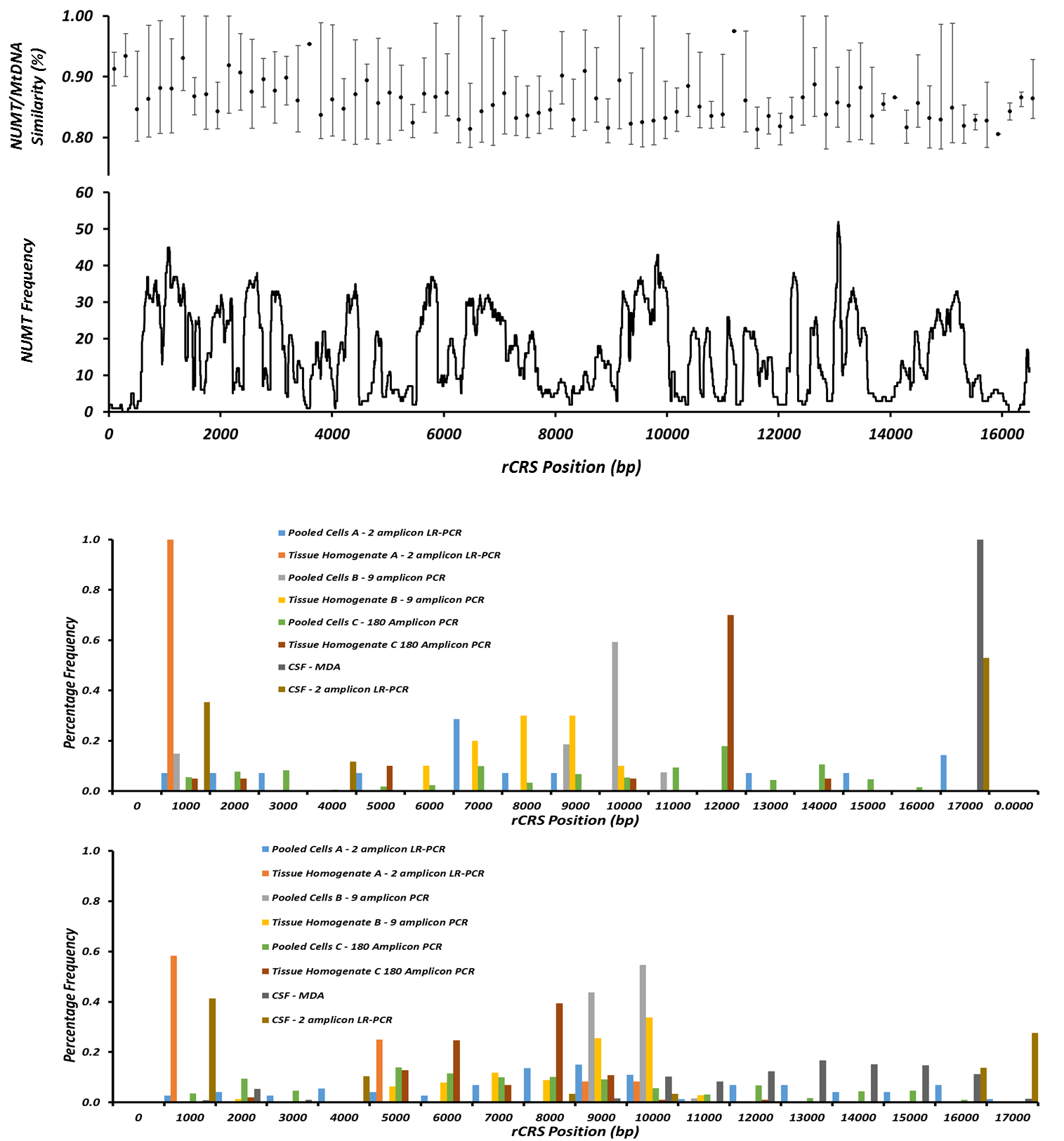
**

**Supplementary Table 1.**  Comparative number of variants identified in each sample cohort (upper panel), detailing the number (and percentage) of each concordant (same, differing by <0.5%), positively shifted (Shift >0.05% towards either rCRS or hg19), "only in rCRS" and "only in hg19" represent variants that were observed only by when one of the references was used (i.e. present at 0.0% using one reference, but >0.0% when using the other). The lower panel shows the number of discrepant variants per category (with percentage per category), when limited to variants below 4% (as in Figure 2). The lower panel demonstrates that, in most instances, rCRS:hg19 variant discrepancies are more prominent when assessing low-level mtDNA heteroplasmy.

| ***Starting Material*** | ***Pooled***  ***Cells A*** | | ***Tissue Homogenate A*** | | ***Pooled***  ***Cells B*** | | ***Tissue Homogenate B*** | | ***Pooled***  ***Cells C*** | | ***Tissue Homogenate C*** | | ***CSF*** | | ***CSF*** | |
| --- | --- | --- | --- | --- | --- | --- | --- | --- | --- | --- | --- | --- | --- | --- | --- | --- |
| ***Sample Number*** | ***28*** | | ***28*** | | ***5*** | | ***15*** | | ***25*** | | ***25*** | | ***3*** | | ***3*** | |
| ***Enrichment*** | ***2 Amplicon*** | | ***2 Amplicon*** | | ***9 Amplicon*** | | ***9 Amplicon*** | | ***180 Amplicons*** | | ***180 Amplicons*** | | ***MDA*** | | ***2 Amplicon*** | |
|  |  |  |  |  |  |  |  |  |  |  |  |  |  |  |  |  |
| ***Heteroplasmy Type*** | ***N*** | ***%*** | ***N*** | ***%*** | ***N*** | ***%*** | ***N*** | ***%*** | ***N*** | ***%*** | ***N*** | ***%*** | ***N*** | ***%*** | ***N*** | ***%*** |
|  |  |  |  |  |  |  |  |  |  |  |  |  |  |  |  |  |
| Same (differing <0.5%) | 2486 | 96.3% | 260 | 93.9% | 79 | 45.4% | 2978 | 63.8% | 46311 | 64.8% | 1172 | 72.7% | 5 | 0.5% | 46 | 43.4% |
|  |  |  |  |  |  |  |  |  |  |  |  |  |  |  |  |  |
| *Shift (>0.5%) to rCRS* | 14 | 0.5% | 2 | 0.7% | 27 | 15.5% | 10 | 0.2% | 1934 | 2.7% | 20 | 1.2% | 3 | 0.3% | 17 | 16.0% |
| *Shift (>0.5%) to hg19* | 5 | 0.2% | 3 | 1.1% | 4 | 2.3% | 1 | 0.0% | 396 | 0.6% | 29 | 1.8% | 1 | 0.1% | 14 | 13.2% |
|  |  |  |  |  |  |  |  |  |  |  |  |  |  |  |  |  |
| *‘only in rCRS’* | 57 | 2.2% | 12 | 4.3% | 64 | 36.8% | 1634 | 35.0% | 19631 | 27.5% | 389 | 24.1% | 967 | 99.1% | 29 | 27.4% |
| *‘only in hg19’* | 20 | 0.8% | 0 | 0.0% | 0 | 0.0% | 48 | 1.0% | 3181 | 4.5% | 1 | 0.1% | 0 | 0.0% | 0 | 0.0% |
|  |  |  |  |  |  |  |  |  |  |  |  |  |  |  |  |  |
|  |  |  |  |  |  |  |  |  |  |  |  |  |  |  |  |  |
|  |  |  |  |  |  |  |  |  |  |  |  |  |  |  |  |  |
|  |  |  |  |  |  |  |  |  |  |  |  |  |  |  |  |  |
| ***Number and percentage of discrepant in each category when limited to <=4% frequency.*** | | | | | | | | | | | | | | | | |
|  |  |  |  |  |  |  |  |  |  |  |  |  |  |  |  |  |
| *Shift (>0.5%) to rCRS* | 6 | 43% | 1 | 50% | 20 | 74% | 10 | 100% | 1874 | 97% | 3 | 15% | 3 | 100% | 14 | 82% |
| *Shift (>0.5%) to hg19* | - | - | - | - | 1 | 25% | 1 | 100% | 250 | 63% | 13 | 45% | - | - | 6 | 43% |
|  |  |  |  |  |  |  |  |  |  |  |  |  |  |  |  |  |
| *‘only in rCRS’* | 57 | 100% | 12 | 100% | 58 | 91% | 1634 | 100% | 17148 | 87% | 360 | 92% | 939 | 97% | 27 | 93% |
| *‘only in hg19’* | 20 | 100% | - | - | - | - | 48 | 100% | 3156 | 99% | - | - | - | - | - | - |
|  |  |  |  |  |  |  |  |  |  |  |  |  |  |  |  |  |

**Supplementary Table 2.**  Spearman correlations of the NUMT frequency per mtDNA base pair and heteroplasmic variant frequency per mtDNA base pair.

| ***Amplification Strategy and Starting Material*** | ***Spearman r*** | ***P value*** |
| --- | --- | --- |
|  |  |  |
| *Pooled Cells A - 2 amplicon LR-PCR* | -0.003 | 7.4x10^-1^ |
| *Tissue Homogenate A - 2 amplicon LR-PCR* | -0.015 | 5.3x10^-2^ |
|  |  |  |
| *Pooled Cells B - 9 amplicon PCR* | -0.023 | 3.2x10^-1^ |
| *Tissue Homogenate B - 9 amplicon PCR* | -0.014 | 6.5x10^-2^ |
|  |  |  |
| *Pooled Cells C - 180 Amplicon PCR* | 0.033 | 7.1x10^-2^ |
| *Tissue Homogenate C 180 Amplicon PCR* | -0.017 | 1.4x10^-1^ |
|  |  |  |
| *CSF - MDA* | 0.063 | 1.0x10^-4^ |
| *CSF - 2 amplicon LR-PCR* | -0.037 | 1.0x10^-4^ |

**Supplementary Table 3.**  Comparison of rCRS/hg19 discrepant heteroplasmies using untrimmed and trimmed data generated using 180 amplicons in pooled cells and tissue homogenate

|  | *Untrimmed* | | *Trimmed* | |  | *Untrimmed* | | *Trimmed* | |
| --- | --- | --- | --- | --- | --- | --- | --- | --- | --- |
|  | ***Pooled Cells*** | | ***Pooled Cells*** | |  | ***Tissue Homogenate*** | | ***Tissue Homogenate*** | |
|  | ***180 Amplicons*** | | ***180 Amplicons*** | |  | ***180 Amplicons*** | | ***180 Amplicons*** | |
| Same (<0.5%) | 54266 | 70.3% | 46311 | 64.8% |  | 4275 | 88.4% | 1172 | 72.7% |
|  |  |  |  |  |  |  |  |  |  |
| Shift (>0.5%) to rCRS | 2297 | 3.0% | 1934 | 2.7% |  | 19 | 0.4% | 20 | 1.2% |
| Shift (>0.5%) to hg19 | 259 | 0.3% | 396 | 0.6% |  | 34 | 0.7% | 29 | 1.8% |
|  |  |  |  |  |  |  |  |  |  |
| ‘only in rCRS’ | 16882 | 21.9% | 19631 | 27.5% |  | 498 | 10.3% | 389 | 24.1% |
| ‘only in hg19’ | 3442 | 4.5% | 3181 | 4.5% |  | 9 | 0.2% | 1 | 0.1% |
|  |  |  |  |  |  |  |  |  |  |
|  | ***Total=77146*** |  | ***Total=71453*** |  |  | ***Total=4835*** |  | ***Total=1611*** |  |
